# Impact of Cooking Techniques on the Dietary Fiber Profile in Selected Cruciferous Vegetables

**DOI:** 10.3390/molecules30030590

**Published:** 2025-01-27

**Authors:** Karolina Nowak, Sascha Rohn, Michał Halagarda

**Affiliations:** 1 Department of Product Packaging, Krakow University of Economics, ul. Rakowicka 27, 31-510 Kraków, Poland; 2 Institute of Food Technology and Food Chemistry, Technische Universität Berlin, Gustav-Meyer-Allee 25, 13355 Berlin, Germany; 3 Department of Food Product Quality, Krakow University of Economics, ul. Sienkiewicza 5, 30-033 Kraków, Poland

**Keywords:** Brassica vegetables, white cauliflower, broccoli, Brussels sprouts, dietary fiber

## Abstract

Cruciferous vegetables of the plant order Brassicales are an attractive dietary component and a valuable source of fiber. However, the nutritional–physiological properties are different when comparing soluble and insoluble fibers. Another significant impact is the transformation of fibers by different influencing factors during food preparation. Cruciferous vegetables, especially, are dominantly processed to soften the matrix. As a result, during cooking, the polysaccharides are dissolved, swelled, or degraded to a certain extent, influencing the composition and the nutritional–physiological properties. The aim of the present study was to analyze the impact of different cooking procedures on changes in the dietary fiber content profile of three different plants: white cauliflower (*Brassica oleracea* L. var. *botrytis*), broccoli (*B. oleracea* L. var. *italica*), and Brussels sprouts (*B. oleracea* L. var. *gemmifera*). The sample material was subjected to direct (“in the water”) and steam cooking. The dietary fiber content and the content of its fractions were determined using an enzymatic analysis method. The results of the research show that the cooking process had a significant influence on the content of dietary fiber fractions in cruciferous vegetables. The concentration of insoluble dietary fiber decreased, whereas the content of soluble dietary fiber increased. When considering the average influence of each process, both steam cooking and direct cooking had a similar impact on changes in the concentrations of dietary fiber fractions. It can therefore be concluded that, when considering dietary fiber content, both processes can be equally well chosen as a thermal treatment for cruciferous vegetables.

## 1. Introduction

Definitions of the term “dietary fiber” have been specified, among several others, by the Codex Alimentarius, the European Food Safety Authority (EFSA), Health Canada, and the U.S. Food and Drug Administration (FDA) [1]. The general definition says that dietary fiber (DF) is the edible part of plants that is not enzymatically digested and adsorbed by the human digestive tract after consumption [2]. DF reaches the colon, where it is either degraded further by the gut microbiome [3] or, after binding to water, simply excreted with the feces [4]. They have positive effects on the human organism by regulating stool weight and intestinal motility, binding to cholesterol or even residues and contaminants, and affecting the homeostasis of nutrients during digestion and absorption [4,5].

DFs belong primarily to the carbohydrate group, as they are dominantly polysaccharides with some modifications. Traditionally, there is a differentiation between two types of DF: water-soluble DF (SDF) and insoluble DF (IDF) [6]. The first type includes, for example, pectins, gums, and mucilages, while the second comprises cellulose, hemicellulose, and lignins [2]. These fractions affect the human organism differently. The viscosity of the chyme affects the form of the stool and the digestive processes, especially the pace of digestion and the absorption of nutrients. SDF increases the viscosity of the chyme, while IDF seems to have only a little effect [4,7]. SDF also facilitates bowel motility, because this fraction forms a gel-like consistency, enabling easier bowel movements [4].

DF in the human diet comes primarily from vegetables, fruits, and grains, as it is obviously a natural component of the cell walls of these plant materials. About 30–40% of the DF in the human diet comes from vegetables [2]. Kalmpourtzidou [8] showed that the consumption of vegetables is insufficient in most countries around the world. Brassica vegetables from the plant order *Brassicales* are an especially attractive dietary component due to their organoleptic characteristics such as taste, smell, and nutritional value, which originate from glucosinolates that are also regarded as very healthy [9]. However, they can be also good sources of DF, as the structure of their stalks and florets is quite rigid and difficult to degrade or digest [10], especially when consumed raw.

Very prominent Brassica vegetables are, among others, broccoli, cauliflower, and Brussels sprouts [9]. These have gained attention because of the proposed positive effect on cancer risk reduction [11]. According to Rodríguez et al. [12], their total DF content can reach up to 28–30% of the dry weight of the vegetables. In the cruciferous vegetables mentioned before, the concentration of total DF ranges between 1 and 4.1 g/100 g of fresh weight [13].

However, the effects of cooking need to be considered, as a certain transformation of DF can be expected. The vegetables are often prepared at home at a higher temperature (boiling, steaming, frying, baking, etc.) [14], which has a general impact on the composition of the vegetables. Some compounds are leached out into the cooking water, while others are transformed into smaller molecules with varying fates [15,16,17,18]. However, the exact mechanisms can be related to the cooking treatments but may also vary, depending on composition and matrix. Cooking methods such as boiling and steaming unlock distinct transformations in the dietary fiber matrix of cruciferous vegetables, potentially enhancing or diminishing their health-promoting properties. These transformations are hypothesized to vary significantly between species due to unique structural compositions [19], unveiling a nuanced interplay between thermal treatment and fiber bioavailability. Therefore, the aim of the present study was to analyze the impact of different cooking procedures on changes in the dietary fiber content profile of selected cruciferous vegetables. For comparing a potential dependency of species, the research material consisted of three different plants: white cauliflower (*Brassica oleracea* L. var. *botrytis*), broccoli (*B. oleracea* L. var. *italica*), and Brussels sprouts (*B. oleracea* L. var. *gemmifera*). The sample material was subjected to direct (“in the water”) and steam cooking. The dietary fiber content and the content of its fractions were determined using an enzymatic analysis method.

## 2. Results and Discussion

The contents of total DF and its fractions, SDF and IDF, in raw, non-processed broccoli, Brussels sprouts, and white cauliflower (Table 1) generally corresponded to the data in the literature. The present values were slightly higher than those obtained by Hedges and Lister [20], Kalala et al. [21], or Lafarga et al. [22] and much higher than those reported by Melim et al. [23] for broccoli, Brussels sprouts, and cauliflower and by Kapusta-Duch et al. [24] for purple cauliflower. At the same time, the present values were slightly lower than those noted by Khanum et al. [25]. Conversely, Núñez-Gómez et al. [26] observed slightly lower DF and IDF contents but a slightly higher SDF concentration. Berndtsson [27] reported slightly higher or lower values depending on the year in which the tests were conducted. When expressed on a fresh matter basis, DF content in cooked broccoli (4.32 g/100 g) was slightly lower and in cauliflower (3.02 g/100 g) slightly higher in this study compared to the values reported by Ferjančič et al. [28] (4.41 g/100 g and 2.8 g/100 g, respectively). Based on the findings described by Wennberg et al. [19], it can be assumed that the discrepancies between the present study’s results and those reported by other authors may be due to differences in the cultivars used. According to Berndtsson [27], differences may be also due to the timepoint during the season.

The research aimed to assess the influence of selected thermal processes on the total dietary fiber (DF) content in cruciferous vegetables, focusing on the impact of plant materials and cooking procedures, as detailed in Table 1. The results showed that, in the case of Brussels sprouts, the total DF content decreased as a result of simple cooking in water. In contrast, an increase in total DF concentration was noted for broccoli and cauliflower. Steam cooking resulted in a significant reduction in total DF in the case of broccoli only. With regard to IDF, a significant decrease was noted for all vegetables tested and both cooking procedures. A difference in processing influence was only significant in the case of Brussels sprouts, where steam cooking had a pronounced effect on IDF concentration. The content of SDF increased significantly in the case of all the tested vegetables. In the case of broccoli, steam cooking had a significantly weaker effect, whereas, in the case of Brussels sprouts, an inverse impact was noted.

When considering the average impact of thermal processes on the three selected vegetables, the type of processing did not have a significant influence on total DF (Table 2). Both steam cooking and water cooking significantly decreased the concentration of IDF (on average, from 34.38 g/100 g to 26.02 g/100 g and 26.3 g/100 g, respectively) and increased the content of SDF (on average, from 2.9 g/100 g to 10.78 g/100 g and 11.8 g/100 g, respectively) in the vegetables tested. According to Ozyurt and Ötle [29], high temperatures during these thermal processes can break down polysaccharides, leading to the release of oligosaccharides, which contributes to the observed increase in SDF. However, in this study, no significant differences between the two analyzed thermal processes were identified.

There is scarce information in the literature on the effect of heat treatment on the content of DF and its two fractions, IDF and SDF, in vegetables. In the present study, a slight but still statistically significant decrease in total DF content was noted as a result of water cooking and, in the case of Broccoli, as a result of steam cooking, too. Considering the average effect on all three tested vegetables, no significant influence on total DF was noted for either of the thermal processes. These results concur with the outcomes described by Nyman et al. [30], which showed that cooking did not significantly affect the DF content of Brussels sprouts. Kalala et al. [21] also found that steaming cauliflower, broccoli, and Brussels sprouts did not significantly affect total DF. However, in the present study, a significant increase in the SDF fraction was observed as a result of both processes. Similarly, Khanum et al. [25], for water-cooked cauliflower, and Kalala et al. [21], for steamed cauliflower, broccoli, and Brussels sprouts, noted a significant increase in SDF fractions. Compared to the results of the present study, the impact was much lower in the case of cauliflower as in the studies described by Kalala et al. [21] or Khanum et al. [25]. At the same time, those authors noted a simultaneous decrease in IDF, a finding that is generally consistent with the results of the present study.

As indicated in Table 1, the reduction in the case of steam-cooked cauliflower was insignificant in the present study. Similarly, Rehman et al. [31] also studied the influence of cooking on its IDF fraction. They showed that, when boiling cauliflower in water, there is a decrease in both neutral detergent fiber and acid detergent fiber. This means that during cooking, these polysaccharidic compounds partially break down. As a result, smaller oligosaccharides and small simple sugars are formed by hydrolysis, which is favored by the heat and the pH conditions during aqueous cooking. Consequently, DF concentrations in the vegetables decrease [31]. Also, according to Khanum et al. [25], the reduction in fiber content in vegetables after cooking may be related to the degradation of IDF. Cellulose is released and also partially degraded further [32]. Steam cooking involves indirect heat transfer through water vapor, which results in a more gentle cooking process and a slower impact on the cellular structures of the vegetables. This may help preserve the integrity of certain dietary fiber fractions, which is reflected in the higher share of IDF fractions in DF compared to water-cooked samples (Table 2). In contrast, Puupponen-Pimiä et al. [33] showed that, in the case of cauliflower, there was an increase in total DF, SDF, and IDF concentrations as a result of blanching combined with freezing. The statistical significance of these results was, however, not verified. Those authors suggested that these increases were due to the mechanical impact of ice crystals on cell wall structures, leading to an improved extraction. However, the mechanical transformation of the cell walls can also force hydrolysis, leading to a better extraction but also to a more probable degradation to smaller compounds.

With regard to nutritional–physiological aspects, nutritionists mostly prefer to maintain IDF in the food [34,35] as its rigidity in structure and complex chemical composition disables a fast breakdown and offers more probability for positively affecting gut passage [36]. Its mechanical stimulation increases fecal bulk, accelerates colonic transit, and alleviates symptoms of constipation, contributing to improved bowel function [37]. IDF is also considered a pre-requisite substrate for the gut microbiome, as it fosters microbial diversity and enhances the release of beneficial secondary metabolites of colonic fermentation, such as short-chain fatty acids (SCFA), particularly butyrate, which exhibit anti-inflammatory properties and support the integrity of the colonic mucosa [3]. These metabolites are vital for maintaining gut health and overall homeostasis, reducing inflammation, and potentially offering protective effects against colorectal diseases. The fermentation of dietary fiber by gut microbiota and the subsequent production of SCFA further underscore the health benefits of maintaining IDF in the diet [38,39]. The findings on fiber transformation during cooking processes highlight key considerations for dietary recommendations. The observed increase in SDF content, along with a decrease in IDF, suggests that cooking methods significantly influence the composition of dietary fiber in vegetables. While IDF is crucial for promoting gut motility and microbiome diversity, SDF provides unique benefits, including enhanced nutrient absorption, the modulation of blood glucose levels, and improved cholesterol metabolism [40]. Individuals should be encouraged to diversify their preparation methods for fiber-rich vegetables like broccoli, cauliflower, and Brussels sprouts. Raw or minimally cooked options minimize fiber degradation and help preserve IDF, supporting intestinal motility and microbiota health, while moderate cooking can increase SDF content, enhancing its effects on metabolic health. Tailoring cooking practices to specific health goals, such as supporting digestive health with higher IDF intake or managing blood sugar levels with increased SDF, could offer personalized nutritional strategies.

**Figure 1:**
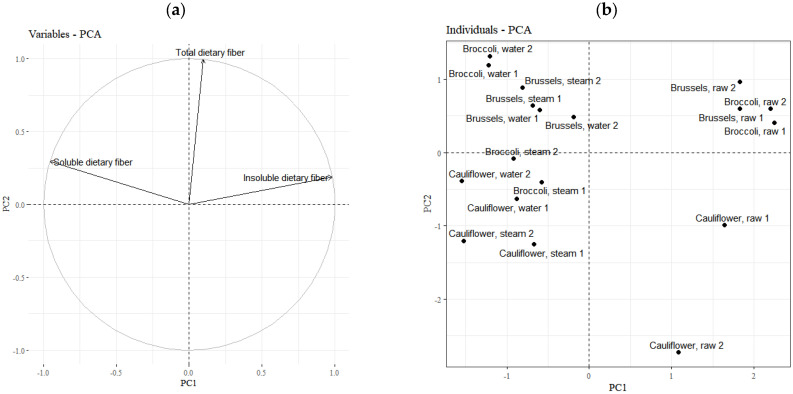
The results of the Principal Component Analysis (PCA). (**a**) The variables factor map; (**b**) The individuals factor map.

In order to illustrate the differences between the selected cruciferous vegetables and their thermal processing on DF and its fractions, as well as water content, PCA was employed (Figure 1). The first two main components received explained 100% of the total variance, with the first component explaining 62.87% and the second explaining 37.13%. As shown in Figure 1a, the first component is associated with a higher IDF/SDF ratio, while the second component corresponds to higher total DF content.

The individual factor map (Figure 1b) shows that the first component separates raw from cooked vegetables. The cooked vegetables had higher IDF/SDF ratios. The second component separates cauliflower from Brussels sprouts and broccoli, indicating that cauliflower has less total DF than the other two vegetables tested. The lack of separation between vegetables subjected to two different thermal processes indicates that steam cooking and water cooking had a similar influence on changes in DF content.

## 3. Materials and Methods

### 3.1. Vegetable Material and Sample Preparation

The research material consisted of white cauliflower (*Brassica oleracea* L. var. *botrytis*), broccoli (*B. oleracea* L. var. *italica*), and Brussels sprouts (*B. oleracea* L. var. *gemmifera*). The vegetables were sourced from a local farmer with a regular harvest in early autumn. To achieve greater homogeneity and ensure consistency in the experiments, the vegetables were inspected to match specific criteria: uniformity in size, color (consistent maturity), and freshness (harvested within 48 h of analysis). Prior to the analysis, the quality was visually inspected to exclude any specimens with visible defects or signs of spoilage. These materials were chosen because they are widely consumed cruciferous vegetables known for their high nutritional value. Furthermore, their varying textures and compositions make them suitable for assessing the effects of different cooking methods on their quality and dietary fiber content.

The sample material was subjected to two methods of heat treatment. One was direct cooking in water, as traditionally carried out very often worldwide. The second procedure is also quite common: steam cooking, with contact with water vapor but a reduced possibility of compounds being leached out. The thermal treatment of vegetables was preceded by the removal of inedible parts and prior washing. In the case of cauliflower and broccoli, the vegetables were additionally divided into florets with a diameter of 7 cm.

The water cooking process was conducted with the use of an induction cooker (Hendi, Hamburg, Germany) and cookware made of stainless steel. Cooking in water was carried out at 100 °C for a time of 12–15 min, depending on the vegetable, until the samples were fully soft. Steam cooking was carried out in an Orange Vision 6× convection steam oven (Retigo, Rožnov pod Radhoštěm, Czech Republic) using full steam. The steaming process lasted 10–12 min, varying by vegetable type, and was terminated when the samples reached full softness. After cooking, the vegetables were cooled to room temperature, frozen at a temperature of −20 ± 2 °C in a shock cooling chamber (RedFox SHS-511, RM Gastro, Veselínad Lužnicí, Czech Republic), freeze-dried using a Christ Alpha 1–4 system (Martin Christ Gefriertrocknungsanlagen GmbH, Osterode am Harz, Germany), and ground in a Tecator Knifetec 1095 laboratory mill (Foss Tecator, Uppsala, Sweden) to obtain a homogeneous material (particle size ≤ 0.5 mm) for further analysis.

### 3.2. Analysis of Total Dietary Fiber, Soluble Dietary Fiber, and Insoluble Dietary Fiber

The dietary fiber content and the content of its fractions were determined using the enzymatic method according to the AOAC 991.43 Standard [41], employing a Total Dietary Fiber Assay Kit (Megazyme, Wicklow, Ireland). While no additional validation experiments were performed for this study, the accuracy of the results was ensured by adhering to the kit’s validated protocols and incorporating reagent blanks to minimize analytical artifacts.

For the purpose of the analysis, 1 g of lyophilized samples were placed in incubation flasks, where the following enzymatic digestion steps were carried out sequentially in a shaking water bath (Memmert WNB14, Buchenbach, Germany):

1. α-Amylase treatment: The sample was suspended in 40 mL of MES-TRIS buffer (pH 8.2) and treated with 50 µL of α-amylase (thermostable). The mixture was incubated at 98–100 °C for 30 min with continuous shaking to solubilize and hydrolyze starch.
2. Protease treatment: After cooling to 60 °C, 100 µL of protease was added to digest proteins. The sample was incubated for 30 min at 60 °C in a shaking water bath.
3. Amyloglucosidase treatment: First, 5 mL of 0.561 M HCl was added, and the pH was adjusted to 4.0–4.6 using 1 M NaOH or 1 M HCl at 60 °C. Subsequently, 300 µL of amyloglucosidase was added while continuously mixing. The flasks were covered with aluminum foil and incubated for 30 min at 60 °C in a shaking water bath to hydrolyze residual starch into glucose.

For the determination of DF, IDF, and SDF, the enzymatic digestate was filtered under vacuum using the Fibertec™ E filtration system (FOSS, Hillerød, Denmark). For IDF, the residue was first rinsed with warm water (2 × 10 mL, 70 °C) to remove soluble components and then sequentially washed with ethanol (78%, 2 × 15 mL; 95%, 2 × 15 mL) and acetone (2 × 15 mL). The filtrates were discarded, and the residues were dried overnight at 105 °C in a Venticell 55 Plus oven dryer (BMT, Brno, Czech Republic). For SDF, the combined filtrate and water washings from the IDF procedure were treated with 230 mL of preheated ethanol (95%, 60 °C) to precipitate soluble fiber. The precipitate was then filtered and subjected to the same rinsing (ethanol and acetone) and drying steps as described for IDF.

Subsequently, the protein content of the residue was determined by the Dumas combustion method [42] using a TruSpec N analyzer (LECO, St. Joseph, MI, USA). A nitrogen-to-protein conversion factor of 5.9 was applied. Ash content was determined by combusting the residue in a muffle furnace (Nabertherm, Lilienthal, Germany) at 525 °C for 5 h [43].

The dietary fiber content (*X*) in g/100 g of the analyzed food product was calculated using the formula:

$$X=\frac{R-A-P-B}{m}\cdot 100,$$

where:

- *R*—mass of the residue after filtration (mg);
- *P*—mass of ash in the sample (mg);
- *A*—mass of protein in the sample (mg);
- *B*—mass of the blank sample residue (mg);
- *m*—mass of the sample (mg).

The results were corrected for moisture content, determined according to ISO 712:2010 [44] using a Venticell 55 Plus oven dryer (BMT, Czech Republic) and expressed as g/100 g on a dry matter basis.

The method’s reliability was verified using the TDF Controls KIT (Megazyme, Wicklow, Ireland). All analyses were performed in triplicate, and reagent blanks were included to account for potential analytical artifacts.

### 3.3. Statistical Analysis

Cooking was repeated four times. For each heat treatment and vegetable type, three replicate samples (1 g each) were analyzed, ensuring reliable measurements and repeatability. To account for variance due to the series of tests, differences between vegetables and types of processing were analyzed with linear mixed models with the series of tests as a random effect and vegetable types and cooking procedures as fixed effects. To compare the influence of thermal processes on selected cruciferous vegetables, a Principal Component Analysis (PCA) was performed. As a result, a two-dimensional sample map was prepared. All the statistical analyses were performed using statistical package R, version 4.4.1 [45]. A value of 0.05 was required for statistical significance.

## 4. Conclusions

The results of this study showed that the cooking process had a significant influence on the content of dietary fiber fractions in selected cruciferous vegetables. When considering the average impact of each process, both steam cooking and water cooking had a similar impact on changes in the concentrations of dietary fiber fractions. It can therefore be concluded that, when considering dietary fiber content, both processes can be equally well chosen as a thermal treatment for the cruciferous vegetables tested. With regard to nutritional–physiological aspects, we would need different tests to decide which processing condition might be preferable. However, to preserve the integrity of insoluble dietary fiber and support digestive health, consumers or the industry may consider raw or minimally cooked vegetables as a preferable option. On the other hand, moderate cooking may increase soluble dietary fiber content, which is beneficial for metabolic health. Tailoring cooking practices to specific health goals, such as maximizing insoluble dietary fiber for gut motility or enhancing soluble dietary fiber for improved metabolic function, could offer personalized dietary strategies.

It is often suggested that steam cooking is more preferential, particularly concerning the retention of water-soluble small molecules such as vitamin C or secondary metabolites like glucosinolates and polyphenols. Ideally, the cooking procedures preserve them as well and lead to a soft, easy-to-consume vegetable matrix while maintaining the quite complex structures of insoluble dietary fiber to the maximum extent. However, future studies should address the balance between preserving fiber content and minimizing nutrient losses in order to make more definitive recommendations.

## Figures and Tables

**Table 1:** Comparison of total dietary fiber content and its insoluble and soluble fractions in broccoli, Brussels sprouts, and white cauliflower with consideration of the effect of thermal processing (g/100 g on a dry matter basis).

Parameter	Vegetable		Type of Processing			Linear Mixed Models		
			Raw	Steam Cooking	Water Cooking	Steam Cooking vs. Raw	Water Cooking vs. Raw	Water Cooking vs. Steam Cooking
Total dietary fiber (DF)	Broccoli	$\bar{x}\pm$ SD	38.72 ± 0.26	36.81 ± 0.4	39.7 ± 0.17	*p* < 0.001 *	*p* < 0.001 *	*p* < 0.001 *
	Brussels sprouts	$\bar{x}\pm$ SD	39.21 ± 0.51	38.8 ± 0.33	38.39 ± 0.09	*p* = 0.059	*p* = 0.001 *	*p* = 0.004 *
	White cauliflower	$\bar{x}\pm$ SD	33.93 ± 2.49	34.81 ± 0.02	36.21 ± 0.27	*p* = 0.291	*p* = 0.017 *	*p* < 0.001 *
Insoluble dietary fiber (IDF)	Broccoli	$\bar{x}\pm$ SD	36.23 ± 0.17	26.38 ± 0.58	26.1 ± 0.09	*p* < 0.001 *	*p* < 0.001 *	*p* = 0.162
	Brussels sprouts	$\bar{x}\pm$ SD	35.22 ± 0.20	27.17 ± 0.14	28.09 ± 0.85	*p* < 0.001 *	*p* < 0.001 *	*p* = 0.007 *
	White cauliflower	$\bar{x}\pm$ SD	31.68 ± 2.17	24.52 ± 1.86	24.73 ± 1.34	*p* < 0.001 *	*p* < 0.001 *	*p* = 0.786
Soluble dietary fiber (SDF)	Broccoli	$\bar{x}\pm$ SD	2.49 ± 0.26	10.43 ± 0.99	13.61 ± 0.08	*p* < 0.001 *	*p* < 0.001 *	*p* < 0.001 *
	Brussels sprouts	$\bar{x}\pm$ SD	3.99 ± 0.32	11.63 ± 0.47	10.31 ± 0.94	*p* < 0.001 *	*p* < 0.001 *	*p* = 0.003 *
	White cauliflower	$\bar{x}\pm$ SD	2.21 ± 0.26	10.29 ± 1.84	11.48 ± 1.61	*p* < 0.001 *	*p* < 0.001 *	*p* = 0.153

* Statistically significant (*p* < 0.05); SD—standard deviation.

**Table 2:** The influence of the selected thermal processing techniques on total dietary fiber content and its insoluble and soluble fractions (g/100 g on a dry matter basis).

Parameter		Process			Linear Mixed Models		
		Raw	Steam Cooking	Water Cooking	Steam Cooking vs. Raw	Water Cooking vs. Raw	Water Cooking vs. Steam Cooking
Total dietary fiber (DF)	$\bar{x}$ ± SD	37.29 ± 2.85	36.81 ± 1.8	38.1 ± 1.59	*p* = 0.825	*p* = 0.7	*p* = 0.447
Insoluble dietary fiber (IDF)	$\bar{x}$ ± SD	34.38 ± 2.35	26.02 ± 1.5	26.3 ± 1.67	*p* = 0.006 *	*p* = 0.009 *	*p* = 0.834
Soluble dietary fiber (SDF)	$\bar{x}$ ± SD	2.9 ± 0.89	10.78 ± 1.16	11.8 ± 1.71	*p* < 0.001 *	*p* = 0.001 *	*p* = 0.39

* Statistically significant (*p* < 0.05); SD—standard deviation.

## Data Availability

The data presented in this study are available on request from the corresponding author.
